# Maternal autoimmune diseases and the risk of tics and Tourette's disorder in offspring: insights from Taiwan's real-world data

**DOI:** 10.3389/fped.2025.1440366

**Published:** 2025-03-04

**Authors:** Yi-Feng Lee, Meng-Che Wu, Yen-Chu Huang, Jing-Yang Huang, James Cheng-Chung Wei

**Affiliations:** ^1^ Division of Neonatology, Children’s Medical Center, Taichung Veterans General Hospital, Taichung, Taiwan; ^2^ School of Medicine, Chung Shan Medical University, Taichung, Taiwan; ^3^ Division of Pediatric Gastroenterology, Children’s Medical Center, Taichung Veterans General Hospital, Taichung, Taiwan; ^4^ Department of Post-Baccalaureate Medicine, College of Medicine, National Chung Hsing University, Taichung, Taiwan; ^5^ Center for Health Data Science, Chung Shan Medical University Hospital, Taichung, Taiwan; ^6^ Institute of Medicine, College of Medicine, Chung Shan Medical University, Taichung, Taiwan; ^7^ Division of Allergy, Immunology and Rheumatology, Chung Shan Medical University Hospital, Taichung, Taiwan; ^8^ Graduate Institute of Integrated Medicine, China Medical University, Taichung, Taiwan

**Keywords:** maternal autoimmune diseases, maternal immune activation, tics and Tourette's disorder, maternal and child health database, Taiwan NHIRD

## Abstract

**Background:**

Currently, tics and Tourette's disorder are burdensome neurological disorders that manifest in vocal and motor tics with onset during childhood. Previous studies have demonstrated that maternal autoimmune diseases may cause several neurodevelopmental disorders in offspring via maternal immune activation. However, the association between them has never been thoroughly researched. Thus, in this study, we aimed to explore whether maternal autoimmune diseases are associated with the risk of tics and Tourette's disorder in offspring in a real-world nationwide population-based cohort study.

**Methods:**

We analyzed offspring with or without autoimmune disease exposure between 2009 and 2016 from national population databases in Taiwan. Multivariate analysis, multiple Cox regression analyses, and stratified analyses were conducted in the study.

**Results:**

In total, 76,411 offspring with autoimmune disease exposure and 1,211,936 offspring without maternal autoimmune disease exposure were selected and analyzed in this study. The incidence of childhood tics and Tourette's disorder was 2.35 [95% confidence interval (CI) 2.23–4.86] and 1.89 (95% CI 1.86–1.92) per 10,000 person-months in children exposed to maternal autoimmune disease and non-exposed children, respectively. The children whose mothers had an autoimmune disease had a 1.26-fold risk of tics and Tourette's disorder compared to children whose mothers did not have an autoimmune disease [crude hazard ratio: 1.26; 95% CI, 1.20–1.34, adjusted hazard ratio (aHR): 1.22; 95% CI, 1.15–1.29]. Offspring of mothers with rheumatoid arthritis (aHR: 1.46, 95% CI, 1.07–1.97), system lupus erythematosus (aHR: 1.57, 95% CI, 1.18–2.09), Sjogren's syndrome (aHR: 1.28, 95% CI, 1.09–1.50), ankylosing spondylitis (aHR: 1.49, 95% CI, 1.07–2.09), Graves’ disease (aHR: 1.26, 95% CI, 1.15–1.37), Hashimoto's thyroiditis (aHR: 1.59, 95% CI, 1.29–1.98), and type I diabetes (aHR: 1.68, 95% CI, 1.13–2.50) had a significantly higher risk of developing tics and Tourette's disorder. Aside from maternal autoimmune diseases, mothers with urinary tract infections, diabetes mellitus, hyperlipidemia, anemia, a sleep disorder, endometriosis, and depression were also associated with childhood tics and Tourette's disorder.

**Conclusion:**

Maternal autoimmune diseases appeared to be associated with tics and Tourette's disorder in offspring, especially in mothers with the abovementioned diseases. Further research is warranted to investigate the possible pathogenetic mechanisms of these associations.

## 1 Introduction

Tics and Tourette's disorder are chronic neurodevelopmental conditions characterized by sudden non-rhythmic motor and vocal tics typically emerging in childhood and persisting for over a year (1). According to the Centers for Disease Control and Prevention (CDC) in the United States, Tourette's disorder affects approximately 0.6% of children, with a three-fold higher prevalence in boys than in girls (2–4). In Taiwan, from 2007 to 2015, the overall incidence of TD increased from 5.34 to 6.87 per 100,000 person-years. Among children and adolescents, the age- and sex-standardized incidence rose from 19.58 to 31.79 per 100,000 person-years, while the prevalence surged from 37.51 per 100,000 people in 2007 to 84.18 per 100,000 in 2015. These trends highlight a growing public health concern (5). Tourette's disorder typically begins between 4 and 10 years of age, significantly affecting children's behavior, academic performance, and social interactions (6, 7). Although hyperkinetic symptoms are mostly ameliorated in adulthood (8), approximately 85% of affected children experience comorbidities such as depression, anxiety, attention deficit hyperactivity disorder (ADHD), autism spectrum disorder, obsessive-compulsive disorder (OCD), and even rage attacks and self-injurious behavior (9). The etiology of Tourette's disorder is multifactorial, including genetic and epigenetic changes, prenatal insults, environmental exposure, emotional stress, and immune-mediated risk factors (10). For example, Tourette's disorder among family members, prenatal emetic drug exposure, assisted reproduction, prenatal or early infections, diet, gut microbiota, sleeping time, exercise, pollution exposure, allergy, and socioeconomic status have been noted as possible risk factors for neurodevelopmental disorders in previous studies (11–16).

Growing evidence suggests that maternal immune activation (MIA) during pregnancy—triggered by infection, autoimmune disease, or dysregulation—may contribute to neurodevelopmental disorders in offspring (17–19). Studies have linked MIA to autism spectrum disorder, ADHD, OCD, schizophrenia, and other behavioral conditions. In addition, maternal autoimmune diseases such as systemic lupus erythematosus (SLE), rheumatoid arthritis, and antiphospholipid syndrome have been associated with higher rates of psychiatric disorders in offspring (20–25). One exception to this rule is represented by maternal multiple sclerosis which is not associated with a higher risk of neurodevelopmental disorders in offspring, perhaps due to the organ-specific characteristic of this central nerve system (CNS) immune-mediated disease (26). Taken together, most of these studies indicate that maternal autoimmune diseases seem to play an important role in the activation of the immune system, through the placenta or circulation, causing neuroinflammatory processes in the fetus and causing neurodevelopmental or mental problems in childhood.

To manage tics and Tourette's disorder, physicians apply behavioral therapies, medication, acupuncture, and neurosurgery such as deep brain stimulation, but the treatment strategy is complex and the response varies (27–31). Recent research has focused on identifying the risk factors and pathophysiological pathways to develop a treatment strategy. Although inflammation is speculated to play a role, evidence also suggests metabolic dysfunction and dysbiosis during early neurodevelopment may contribute (11, 32–35). However, the exact maternal-fetal pathway remains unclear. A 2016 review established a positive bidirectional association between autoimmune diseases and OCD and tic disorders (36). However, few epidemiological research studies have explored maternal autoimmune diseases as a risk factor (23, 37–39). We hypothesized that maternal autoimmune diseases, through immune-inflammatory activation processes, increase the risk of tics and Tourette's disorder in offspring and conducted a population-based, nationwide cohort study using Taiwan's National Health Insurance Research Database (NHIRD).

## 2 Materials and methods

### 2.1 Data source

We retrieved the family health history for multiple generations and the relationship between children and their birth parents by linking the 2009–2019 NHIRD, Birth Registration Database, Maternal and Child Health Database, and the National Death Index Database (40), which are regulated by the Health and Welfare Data Science Center (HWDC) in Taiwan. The NHIRD contains all outpatient and inpatient medical claims, including drug medications, medical operations, procedures, and fees. The Birth Registration Database contains birth weight, gestational weeks, delivery type, live birth, stillbirth, multiple births, and nationality of the mother. The Maternal and Child Health Database contains the parent's and child's de-identified numbers. By linking these databases, we could trace each mother's comorbidities and medications during pregnancy. Our study was approved by the Institutional Review Board of Chung Shan Medical University Hospital (approval no. CS2-21006).

### 2.2 Study group and outcome measurement

This study was a retrospective cohort study design. We retrieved individuals from the birth certificate application database from 2009 to 2016. The following were excluded: mother's identification missing, nationality missing, foreign nationality, multiple births, and stillbirth. The exposure group was offspring with autoimmune disease exposure. The definition was a diagnosis of autoimmune disease (including rheumatoid arthritis, system lupus erythematosus, Sjogren's syndrome, ankylosing spondylitis, psoriasis, Graves’ disease, Hashimoto's thyroiditis, polyarteritis nodosa, uveitis, inflammatory bowel diseases, and type I diabetes, which were coded as follows: International Classification of Diseases, 9th Revision, Clinical Modification (ICD-9-CM) codes 714, 710, 710.2, 720, 696, 696.1, 242, 245.2, 446, 360.12, 363.0x, 363.1x, 363.20, 363.21, 363.22, 364.0x, 364.1x, 364.2x, 364.3, 555, 556, and 250.01; ICD-10-CM code M05, M06, M07, M09, M32, M35, M45, L40, E05, E06.3, M30, H44.11, H30, H20, K50, K51, and E10) in the mother during pregnancy or 1 year before pregnancy. The comparison group was the offspring of mothers never diagnosed with an autoimmune disease during pregnancy or 1 year before pregnancy. The index date was set as the birth date.

The outcome variable was defined as a diagnosis of tics and Tourette's disorder (ICD-9-CM code: 307.2, 37.20, 37.21, 37.22, 37.23; ICD-10-CM code: F95.0, F95.1, F95.2, F95.8, F95.9) during the observational period in NHIRD. Patients seeking medical care exhibiting symptoms or such abnormal behaviors, who had three outpatient department visits or one admission record with any type of tic or Tourette's disorder, were enrolled as the outcome in this study. Both groups, offspring of mothers with autoimmune diseases or without, were followed up until the onset of tics and Tourette's disorder, death, or 31 December 2019, whichever occurred first.

### 2.3 Covariates and matching

The baseline characteristics were birth year, child's sex, birth weight (<2,500, 2,500–3,499, ≥3,500 g), gestational weeks (<36, 36–40, ≥41 weeks), delivery [normal spontaneous delivery, NSD, cesarean section (C/S)], parity, parents’ ages, congenital defects, urbanization, insurance unit, maternal and paternal comorbidities, children or parents died within 1 year after birth, medication exposure during pregnancy, and maternal comorbidities, including asthma (ICD-CM = 493, J44, J45), hypertension (ICD-CM = 401–405, I10–I15), diabetes mellitus (ICD-CM = 250, E10, E11, E12, E13, E14), hyperlipidemia (ICD-CM = 272, E78), gestational diabetes (ICD-CM = 648.8, O99.81, O24.41, O24.42, O24.43), preeclampsia or eclampsia (ICD-CM = 6,424.4, 642.5, 642.6, 642.7, O11, O14,O15), cancer (ICD-CM = 140–199, 200–208, C00–C97), urinary tract infection (ICD-CM = 599, N39), anemia (ICD-CM = 281–285, D60–D64), endometriosis (ICD-CM = 617.0–617.9, 621.3, N80), sleep disorder (ICD-CM = 327, 780.5, G47), depression (ICD-CM = 293.83, 296.2, 296.3,300.4, 311, F06.3, F32.0, F32.1, F32.2, F32.3, F32.4, F32.5, F32.9, F33.0, F33.1, F33.2, F33.3, F33.4, F33.9, F34.1), postpartum depression (ICD-CM = 648.44, F53), and seizure disorder (ICD-CM = 345, G40–41). In addition, paternal comorbidities, including asthma, hypertension, diabetes mellitus, hyperlipidemia, cancers, urinary tract infection, anemia, sleep disorder, depression, and seizure disorder, were also considered for further multivariate analysis. These parental comorbidities were defined within 2 years before the child’s birth.

### 2.4 Statistical analysis

Comparisons between the maternal autoimmune disease cohort and the non-maternal autoimmune disease cohort were performed using absolute standardized difference (ASD). The characteristics of the groups were considered balanced when the ASD value was less than 0.1 (41). The incidence density was calculated as the number of newly diagnosed cases per 10,000 person-months. The confidence intervals of incidence density were calculated using the Rothman–Greenland method (42). Kaplan–Meier analysis was used to calculate the 10-year cumulative incidence of tics and Tourette's disorder between the two cohorts. The log-rank test was used to test the significance. To determine the independent risk of the maternal autoimmune disease group, a multivariable Cox proportional hazard model was used to estimate the adjusted hazard ratios (HRs). The propensity score-matched (PSM) cohorts were selected to further balance potential confounders between the two study cohorts (41). The propensity score (probability) of maternal autoimmune disease exposure was calculated using logistic regression, incorporating covariates such as baseline perinatal characteristics, including the child’s birth year, child’s sex, delivery method, parity, parental age, urbanization, insurance coverage, comorbidities, and aspirin exposure during pregnancy. Subsequently, each child with maternal autoimmune disease exposure was matched with two non-exposed children with a similar propensity score, achieved through the nearest neighbor greedy algorithm with a caliper of 0.01. However, PSM still fails to address unmeasured confounding effects, and its application when selecting the study population is associated with numerous limitations and potential biases (43). In studies involving a large sample size, the traditional covariate adjustment method performs similarly to the propensity score methods, which include propensity score stratification, propensity score matching, and propensity score inverse probability weighting (44). Therefore, we based our primary results on the traditional covariate adjustment method. In addition, we performed stratified analyses to observe the HRs within different subgroups. The statistical software used for the analyses was SAS version 9.4 (SAS Institute Inc., NC, USA).

## 3 Results

### 3.1 Study population and cohort selection

**Figure 1:**
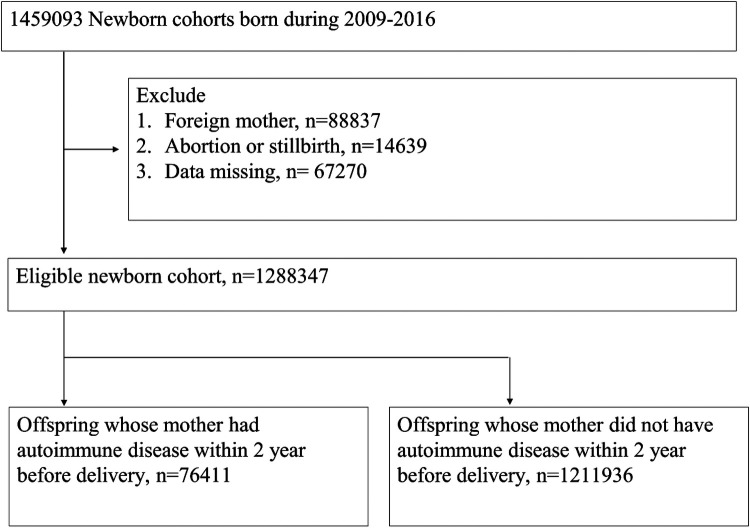
Flowchart of the study groups.

In total, 1,459,093 individuals were retrieved from the birth certificate applications database from 2009 to 2016 and 1,288,347 offspring passed the exclusion criteria. We selected 76,411 offspring with maternal autoimmune disease exposure and 1,211,936 individuals without maternal autoimmune disease exposure for analysis (Figure 1).

### 3.2 Demographic characteristics

Among the demographic and perinatal characteristics of both cohorts, the maternal autoimmune disease exposure cohort had higher prevalence rates of maternal comorbidities, such as diabetes mellitus (4.71% vs. 2.56%, with ASD = 0.115), hyperlipidemia (1.63% vs. 0.45%, with ASD = 0.116), anemia (17.82% vs. 8.4%, with ASD = 0.282), sleep disorder (7.15% vs. 4.45%, with ASD = 0.116), and depression (4.62% vs. 2.68%, with ASD = 0.103), and higher proportions of aspirin, hydroxychloroquine, azathioprine, or sulfasalazine users when compared with those in the without maternal autoimmune disease exposure group. The other comorbidities and risk factors, including preterm or low birth weight, birth year, child's sex, birth weight, delivery mode, parity, Apgar score, congenital defects, urbanization, and insurance unit showed no difference between the maternal autoimmune disease exposure and without maternal autoimmune exposure groups (all ASD < 0.1). We used propensity score matching to balance the baseline perinatal characteristics, including the child’s birth year, child’s sex, delivery method, parity, parental age, urbanization, insurance coverage, comorbidities, and aspirin exposure during pregnancy between the two study cohorts (Table 1).

**Table 1:** Demographic and perinatal characteristics of the non-exposure cohort and maternal autoimmune disease exposure cohort.

Variables	Before PSM			After PSM		
	Non-exposure cohort	Maternal autoimmune disease exposure cohort	ASD	Non-exposure cohort	Maternal autoimmune disease exposure cohort	ASD
Number	1,211,936	76,411		141,776	70,888	
Offspring factors						
Birth year						
2009	44,980 (3.71%)	2,604 (3.41%)	0.016	4,899 (3.46%)	2,464 (3.48%)	0.001
2010	130,097 (10.73%)	7,557 (9.89%)	0.028	14,060 (9.92%)	7,062 (9.96%)	0.002
2011	158,534 (13.08%)	9,477 (12.40%)	0.020	17,710 (12.49%)	8,855 (12.49%)	0.000
2012	179,307 (14.80%)	11,457 (14.99%)	0.006	21,469 (15.14%)	10,723 (15.13%)	0.001
2013	167,003 (13.78%)	10,896 (14.26%)	0.014	20,222 (14.26%)	10,145 (14.31%)	0.001
2014	172,777 (14.26%)	11,056 (14.47%)	0.006	20,544 (14.49%)	10,237 (14.44%)	0.001
2015	183,655 (15.15%)	11,954 (15.64%)	0.014	21,976 (15.50%)	11,016 (15.54%)	0.001
2016	175,583 (14.49%)	11,410 (14.93%)	0.013	20,896 (14.74%)	10,386 (14.65%)	0.003
Sex						
Male	628,654 (51.87%)	39,460 (51.64%)	0.005	73,332 (51.72%)	36,622 (51.66%)	0.001
Female	583,282 (48.13%)	36,951 (48.36%)	0.005	68,444 (48.28%)	34,266 (48.34%)	0.001
Gestational weeks						
<37	109,330 (9.02%)	8,947 (11.71%)	0.088	14,001 (9.88%)	7,763 (10.95%)	0.035
37–41	1,074,652 (88.67%)	65,797 (86.11%)	0.077	124,689 (87.95%)	61,566 (86.85%)	0.033
≥41	27,954 (2.31%)	1,667 (2.18%)	0.008	3,086 (2.18%)	1,559 (2.20%)	0.002
Birth weight (g)						
<2,500	103,250 (8.52%)	8,403 (11.00%)	0.084	13,136 (9.27%)	7,208 (10.17%)	0.031
2,500–3,500	939,683 (77.54%)	58,044 (75.96%)	0.037	109,034 (76.91%)	54,170 (76.42%)	0.012
≥3,500	169,003 (13.94%)	9,964 (13.04%)	0.027	19,606 (13.83%)	9,510 (13.42%)	0.012
Apgar score <5						
At 1 min	6,166 (0.51%)	586 (0.77%)	0.032	768 (0.54%)	494 (0.70%)	0.020
At 5 min	1,107 (0.09%)	111 (0.15%)	0.016	138 (0.10%)	92 (0.13%)	0.010
Congenital defects	32,421 (2.68%)	2,491 (3.26%)	0.035	3,864 (2.73%)	2,258 (3.19%)	0.027
Death within 1 year of birth	2,663 (0.22%)	197 (0.26%)	0.008	306 (0.22%)	163 (0.23%)	0.003
Maternal factors						
Age (years)						
<30	392,064 (32.35%)	22,366 (29.27%)	0.067	41,672 (29.39%)	20,960 (29.57%)	0.004
30–39	781,857 (64.51%)	51,295 (67.13%)	0.055	95,540 (67.39%)	47,573 (67.11%)	0.006
≥40	38,015 (3.14%)	2,750 (3.60%)	0.026	4,564 (3.22%)	2,355 (3.32%)	0.006
Urbanization						
Urban	776,238 (64.05%)	49,041 (64.18%)	0.003	91,291 (64.39%)	45,525 (64.22%)	0.004
Sub-urban	368,671 (30.42%)	23,532 (30.80%)	0.008	43,739 (30.85%)	21,879 (30.86%)	0.000
Rural	67,027 (5.53%)	3,838 (5.02%)	0.023	6,746 (4.76%)	3,484 (4.91%)	0.007
Insurance unit						
Government	80,619 (6.65%)	5,527 (7.23%)	0.023	10,251 (7.23%)	5,167 (7.29%)	0.002
Labor	930,967 (76.82%)	59,397 (77.73%)	0.022	112,735 (79.52%)	55,940 (78.91%)	0.015
Agricultural/fisherman/water resources employee	52,504 (4.33%)	3,112 (4.07%)	0.013	5,630 (3.97%)	2,904 (4.10%)	0.006
Low-income	3,154 (0.26%)	220 (0.29%)	0.005	204 (0.14%)	116 (0.16%)	0.005
Non-labor force	109,244 (9.01%)	5,807 (7.60%)	0.051	8,674 (6.12%)	4,567 (6.44%)	0.013
Others	35,448 (2.92%)	2,348 (3.07%)	0.009	4,282 (3.02%)	2,194 (3.10%)	0.004
Delivery methods						
NSD	773,532 (63.83%)	45,678 (59.78%)	0.083	85,699 (60.45%)	42,750 (60.31%)	0.003
C/S	438,404 (36.17%)	30,733 (40.22%)	0.083	56,077 (39.55%)	28,138 (39.69%)	0.003
Parity						
Singleton	1,172,925 (96.78%)	73,204 (95.80%)	0.052	136,408 (96.21%)	67,952 (95.86%)	0.018
Multiparity	39,011 (3.22%)	3,207 (4.20%)	0.052	5,368 (3.79%)	2,936 (4.14%)	0.018
Comorbidity while pregnancy						
Asthma	13,103 (1.08%)	1,128 (1.48%)	0.035	1,872 (1.32%)	993 (1.40%)	0.007
Hypertension	18,113 (1.49%)	2,058 (2.69%)	0.084	3,513 (2.48%)	1,806 (2.55%)	0.005
Diabetes mellitus	31,062 (2.56%)	3,599 (4.71%)	0.115	6,355 (4.48%)	3,338 (4.71%)	0.011
Hyperlipidemia	5,510 (0.45%)	1,247 (1.63%)	0.116	2,038 (1.44%)	1,110 (1.57%)	0.011
Malignancy	3,608 (0.30%)	478 (0.63%)	0.048	854 (0.60%)	435 (0.61%)	0.002
Urinary tract infection	145,187 (11.98%)	11,212 (14.67%)	0.079	20,608 (14.54%)	10,274 (14.49%)	0.001
Seizure disorder	1,498 (0.12%)	145 (0.19%)	0.017	237 (0.17%)	124 (0.17%)	0.002
Anemia	101,787 (8.40%)	13,617 (17.82%)	0.282	25,944 (18.30%)	12,577 (17.74%)	0.015
Gestational diabetes	79,385 (6.55%)	5,590 (7.32%)	0.030	10,485 (7.40%)	5,233 (7.38%)	0.001
Eclampsia or preeclampsia	44,373 (3.66%)	3,941 (5.16%)	0.073	7,003 (4.94%)	3,582 (5.05%)	0.005
Endometriosis	13,462 (1.11%)	1,138 (1.49%)	0.033	2,011 (1.42%)	1,042 (1.47%)	0.004
Sleep disorder	53,983 (4.45%)	5,466 (7.15%)	0.116	9,934 (7.01%)	4,897 (6.91%)	0.004
Depression	32,540 (2.68%)	3,530 (4.62%)	0.103	6,320 (4.46%)	3,072 (4.33%)	0.006
Postpartum depression	50,002 (4.13%)	4,777 (6.25%)	0.096	8,323 (5.87%)	4,217 (5.95%)	0.003
Medications during pregnancy						
Aspirin	26,741 (2.21%)	3,834 (5.02%)	0.151	4,496 (3.17%)	2,444 (3.45%)	0.016
Hydroxychloroquine	894 (0.07%)	2,850 (3.73%)	0.270	155 (0.11%)	1,850 (2.61%)	0.217
Methotrexate	63 (0.01%)	130 (0.17%)	0.056	11 (0.01%)	110 (0.16%)	0.052
Azathioprine	175 (0.01%)	598 (0.78%)	0.122	29 (0.02%)	287 (0.41%)	0.084
Cyclosporin	34 (0.00%)	128 (0.17%)	0.057	6 (0.00%)	89 (0.13%)	0.048
Sulfasalazine	61 (0.01%)	520 (0.68%)	0.116	10 (0.01%)	400 (0.56%)	0.105
Death within 1 year of delivery	338 (0.03%)	47 (0.06%)	0.016	65 (0.05%)	34 (0.05%)	0.001
Paternal factors	*n* = 1,155,140	*n* = 73,296		*n* = 140,397	*n* = 70,146	
Age (years)						
<30	224,320 (19.42%)	12,829 (17.50%)	0.049	24,800 (17.49%)	12,524 (17.67%)	0.005
30–39	797,507 (69.04%)	51,553 (70.34%)	0.028	99,163 (69.94%)	49,271 (69.51%)	0.009
≥40	133,313 (11.54%)	8,914 (12.16%)	0.019	16,434 (11.59%)	8,351 (11.78%)	0.006
Comorbidity						
Asthma	16,779 (1.45%)	1,111 (1.52%)	0.005	1,937 (1.37%)	1,064 (1.50%)	0.012
Hypertension	35,267 (3.05%)	2,564 (3.50%)	0.025	4,596 (3.24%)	2,426 (3.42%)	0.010
Diabetes mellitus	14,007 (1.21%)	1,087 (1.48%)	0.024	1,873 (1.32%)	1,040 (1.47%)	0.013
Hyperlipidemia	39,942 (3.46%)	3,169 (4.32%)	0.045	5,785 (4.08%)	3,000 (4.23%)	0.008
Malignancy	4,352 (0.38%)	283 (0.39%)	0.002	473 (0.33%)	263 (0.37%)	0.006
Urinary tract infection	13,681 (1.18%)	1,004 (1.37%)	0.017	1,841 (1.30%)	961 (1.36%)	0.005
Seizure disorder	1,674 (0.14%)	111 (0.15%)	0.002	173(0.12%)	108(0.15%)	0.008
Sleep disorder	55,072(4.77%)	4,057(5.54%)	0.035	7,430(5.24%)	3,854(5.44%)	0.009
Depression	33,833(2.93%)	2,537(3.46%)	0.030	4,597(3.24%)	2,426(3.42%)	0.010
Death within 1 year of delivery	809(0.07%)	48(0.07%)	0.002	56(0.04%)	44(0.06%)	0.010

NSD, normal spontaneous delivery; C/S, cesarean section.

Using propensity PSM, we matched the maternal autoimmune disease exposure cohort with the non-exposure cohort based on potential confounders, such as baseline perinatal characteristics, including the child’s birth year, child’s sex, delivery method, parity, parental age, urbanization, insurance coverage, comorbidities, and aspirin exposure during pregnancy.

### 3.3 Incidence of tics and Tourette's disorder

Table 2 displays the incidence rates per 10,000 person-months of childhood tics and Tourette's disorder before propensity score matching. The maternal autoimmune disease exposure cohort exhibited a significantly higher incidence rate (2.35, 95% CI = 2.23–2.48) compared to the non-exposure cohort (1.89, 95% CI = 1.86–1.92).

**Table 2:** Risk of tics and Tourette's disorder in offspring exposed to maternal autoimmune diseases.

	Before PSM		After PSM	
	Non-exposure	Maternal autoimmune disease	Non-exposure	Maternal autoimmune disease
*n*	1,211,936	76,411	141,776	70,888
Observed person-months	92,966,269	5,785,391	10,780,509	5,385,379
Event of tics and Tourette's disorder	17,543	1,359	2018	1,263
Incidence rate (95% CI)	1.89 (1.86–1.92)	2.35 (2.23–2.48)	1.87 (1.79–1.96)	2.35 (2.22–2.48)
Crude hazard ratio (95% CI)	Reference	1.26 (1.20–1.34)	Reference	1.25 (1.17–1.35)
aHR1 (95% CI)	Reference	1.22 (1.15–1.29)		
aHR2 (95% CI)	Reference	1.21 (1.15–1.29)		

The incidence rate is per 10,000 person-months; the 95% CI was estimated using the Rothman–Greenland method.

aHR1 denotes the hazard ratio for tics and Tourette's disorder in children exposed to maternal autoimmune disease adjusted for covariates including the child’s birth year, child’s sex, delivery method, parity, urbanization, insurance coverage, maternal age, maternal comorbidities (such as asthma, hypertension, diabetes, hyperlipidemia, malignancy, urinary tract infection, seizure disorder, anemia, eclampsia or preeclampsia, endometriosis, sleep disorder, and depression), maternal aspirin exposure, paternal age, and paternal comorbidities (including asthma, hypertension, diabetes, hyperlipidemia, malignancy, urinary tract infection, seizure disorder, sleep disorder, and depression) during pregnancy. aHR2 denotes the hazard ratio for tics and Tourette's disorder in children exposed to maternal autoimmune adjusted for the covariates of aHR1 and additional factors including gestational weeks, birth weight, Apgar score < 5 at 1 min, and child congenital defects. PSM denotes the propensity score matching that was employed to select a maternal autoimmune disease exposure cohort paired with a non-exposure cohort. This pairing was based on potential confounders, including baseline perinatal characteristics such as the child’s birth year, child’s sex, delivery method, parity, urbanization, insurance coverage, parental age, parental comorbidities, and maternal aspirin exposure during pregnancy.

### 3.4 Cumulative probability of tics and Tourette's disorder

**Figure 2:**
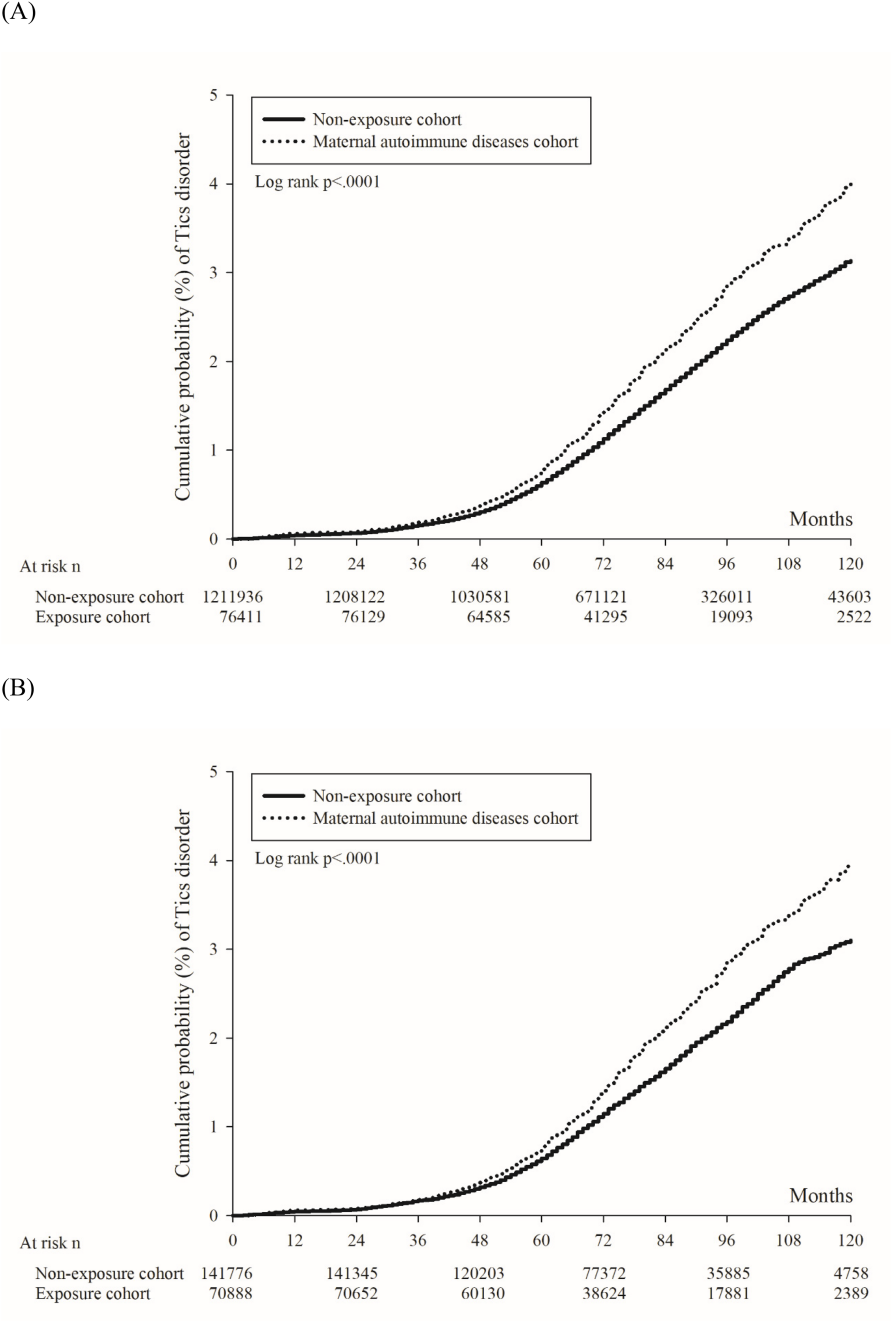
Kaplan–Meier curve of the cumulative probability of tics and Tourette's disorder in offspring. **(A)** Before propensity score matching. **(B)** After propensity score matching.

In Figure 2A, the 10-year cumulative probability of tics and Tourette's disorder was higher in the maternal autoimmune disease exposure cohort (4.00%) than in the non-exposure cohort (3.13%), with the log-rank test yielding a *p*-value of <0.001. The unadjusted hazard ratio for tics and Tourette's disorder was 1.26 (95% CI = 1.20–1.34) in children born to mothers with autoimmune disease, in comparison to those born to mothers without autoimmune disease. After adjusting for perinatal characteristics, the adjusted hazard ratio (aHR1, the model recommended by experts) was calculated as 1.22 (95% CI = 1.15–1.29), and aHR2 (the full model) was determined to be 1.21 (95% CI = 1.15–1.29) (Table 2). Following propensity score matching, the hazard ratio was found to be 1.25 (95% CI = 1.17–1.35), and the 10-year cumulative probability of tics and Tourette's disorder within the propensity score-matched cohorts is illustrated in Figure 2B.

### 3.5 Risk stratification by maternal and child factors

**Figure 3:**
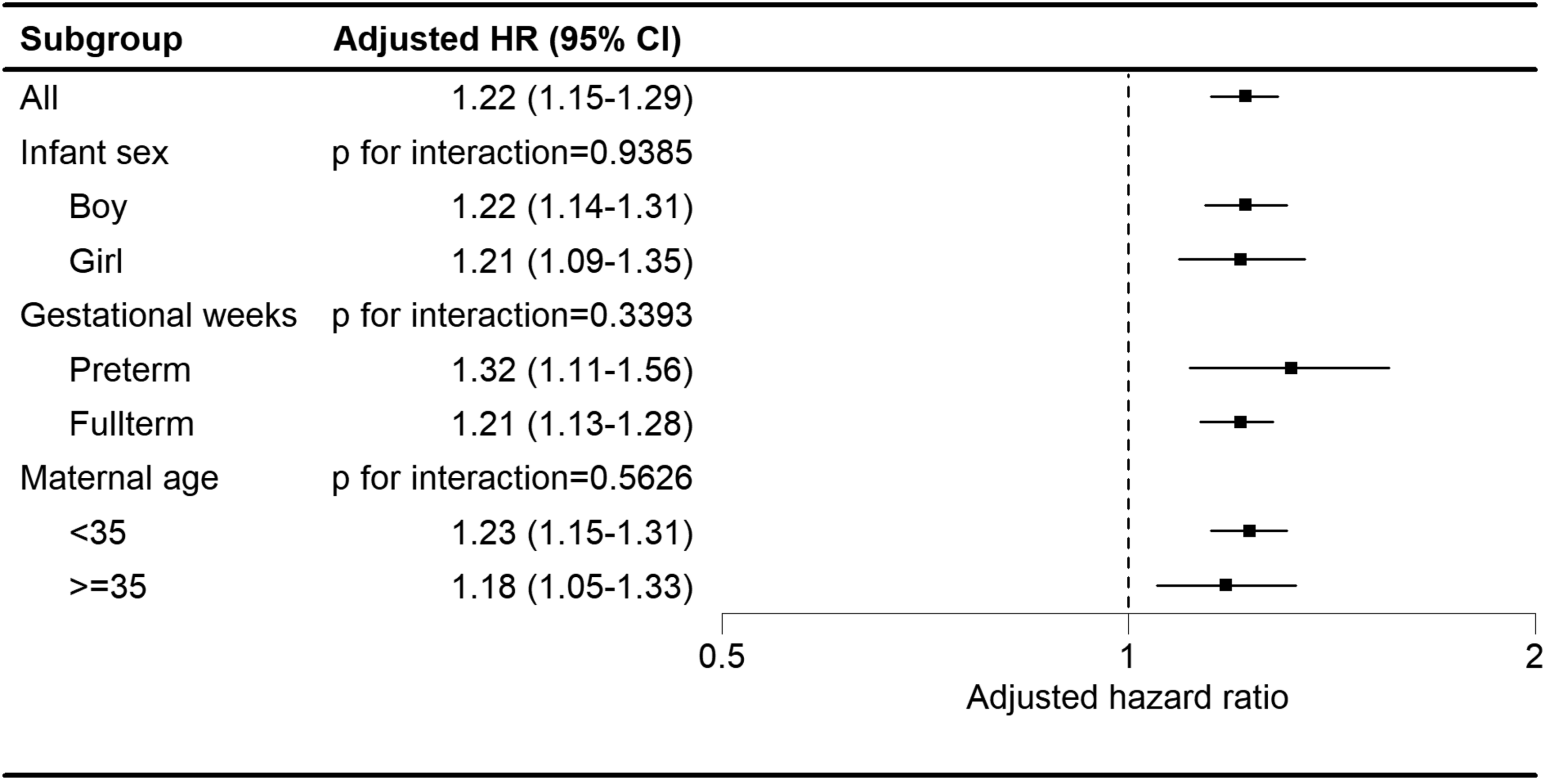
Subgroup analysis of the association between maternal autoimmune disease and development of tics and Tourette's disorder in offspring.

Regardless of the child's sex, gestational age, or maternal age strata, children in the maternal autoimmune disease exposure cohort showed an elevated likelihood of developing tics and Tourette's disorder (Figure 3).

### 3.6 Impact of specific maternal autoimmune diseases

**Figure 4:**
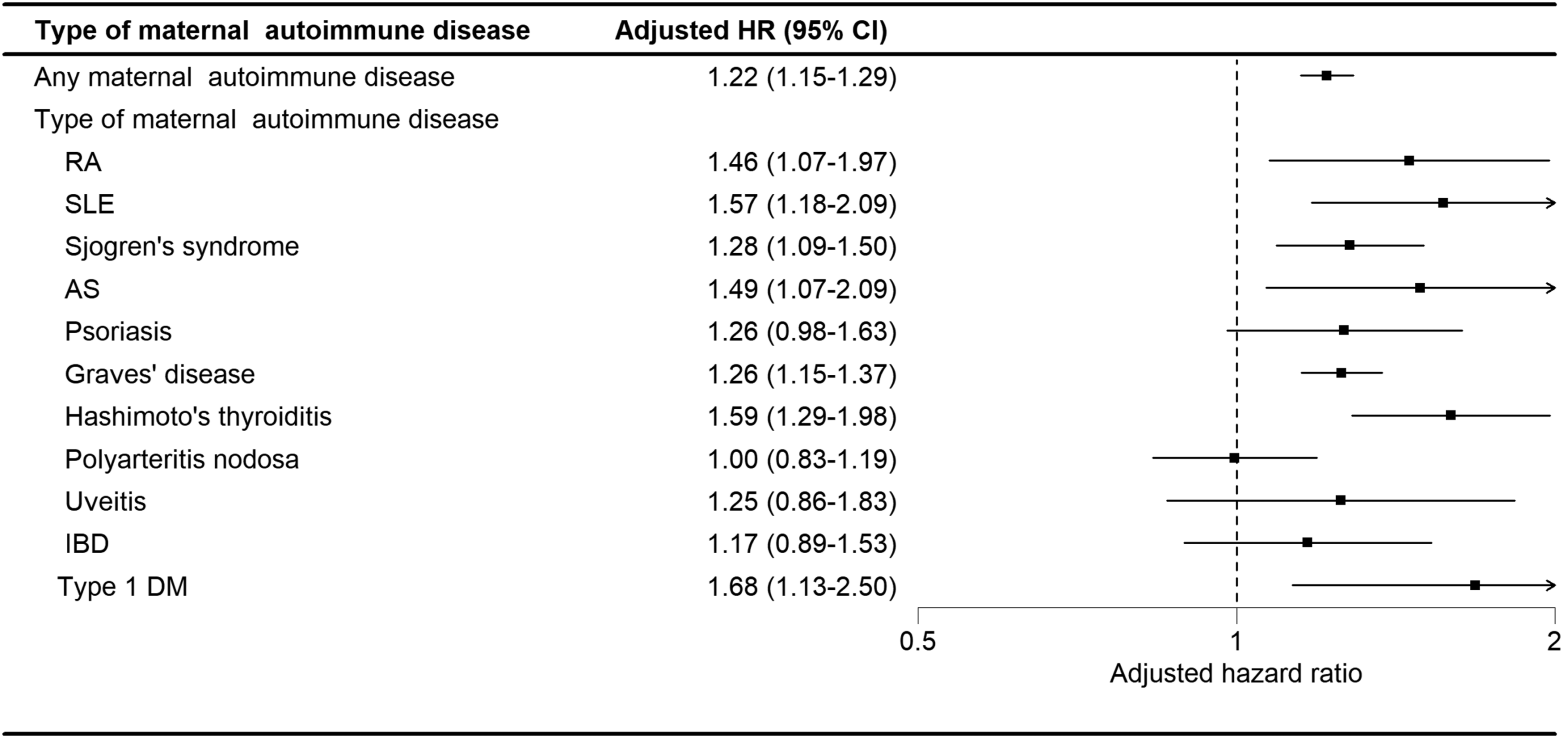
Adjusted HRs for tics and Tourette's disorder by subtype of maternal autoimmune disease.

As shown in Figure 4, in the analysis stratified by the type of maternal autoimmune disease exposure, offspring exposed to maternal rheumatoid arthritis (aHR: 1.46, 95% CI, 1.07–1.97), systemic lupus erythematosus (aHR: 1.57, 95% CI, 1.07–1.97), Sjogren's syndrome (aHR: 1.28, 95% CI, 1.09–1.50), ankylosing spondylitis (aHR: 1.49, 95% CI, 1.07–2.09), Graves’ disease (aHR: 1.26, 95% CI, 1.15–1.37), Hashimoto's thyroiditis (aHR: 1.59, 95% CI, 1.29–1.98), or type I diabetes (aHR: 1.68, 95% CI, 1.13–2.50) had a significantly elevated risk of developing tics and Tourette's disorder.

## 4 Discussion

To the best of our knowledge, this study is the first to analyze a large, nationwide cohort using the Maternal and Child Health Database to explore the link between maternal autoimmune disorders and childhood tics and Tourette's disorders in an Asian population. We found a significantly higher incidence and cumulative risk of these neurobehavior disorders in children born to mothers with autoimmune diseases, especially rheumatoid arthritis, systemic lupus erythematosus, Sjogren's syndrome, ankylosing spondylitis, Graves’ disease, Hashimoto's thyroiditis, and type I diabetes. These findings highlight the importance of early monitoring for neurodevelopmental issues in children of affected mothers.

The etiology of tics and Tourette's disorder is multifactorial, involving multiple genetic (45, 46), environmental, or immunological factors. Studies have suggested that autoimmune diseases may lead to excessive immune response, abnormal cytokine levels, dysregulation of neurotransmitters, and microglial dysfunction, potentially disrupting fetal brain development. While these mechanisms are well-studied in animal models and neuroimage and postmortem analyses, direct evidence in humans remains limited (47–53). Children with tics and Tourette's disorder sometimes have a sensory premonitory urge prior to the onset of the motor component (6), implying that neurotransmitter dysregulation, including gamma-aminobutyric acid (GABA), glutamate, and dopamine pathways, is involved in neuroinflammation. For example, the generation of unplanned movement or sounds is associated with disruption of GABA-related disinhibition or poor-regulated glutamate excitation in the basal ganglia and dopaminergic system dysregulation in the substantia nigra (54, 55).

Maternal autoimmune diseases may cause excessive immune-cellular responses, dysregulated serum cytokines, and poor long-lasting imprinting of the immune system, especially during pregnancy (56–58). The MIA, via a dysfunctional and inflammatory intrauterine phenomenon, may lead to neuroinflammation in fetal brain development or early childhood, leading to neurodevelopmental disease (16, 39, 59, 60). For example, microglial cells were believed to be involved in OCD, Tourette’s disorder, pediatric autoimmune neuropsychiatric disorders associated with streptococcal infections (PANDAS), or psychiatric disease (19, 61–65). Abnormal cytokines, such as monocyte chemotactic factor-1 (MCP-1), interleukin-2 (IL-2), and protein tyrosine phosphatase receptor-N (PTPR-N) were noted in patients with Tourette’s disorder (66). In a recent animal model study, alpha-enolase-specific antibody, which is often detected in autoimmune disease, was found to be highly expressed in the maternal circulation and in the brain tissues of offspring, causing impaired learning and memory abilities through complement-dependent cytotoxicity (67). These pathophysiological mechanisms have been well-studied in animal models, adults, and postmortem studies, indicating that abnormal immunity promotes the development of neurogenic disease, but there is still limited evidence of transfer from the mother across the placenta to the fetal brain in human beings, manifesting as a neurogenic disorder in early childhood, the developmental stage in which the onset of tics and Tourette's disorder occurs. It is worth mentioning that a recent study presents a new concept of neurodevelopmental disorders in the case of autism. Sotgiu et al. (65) indicated that, aside from altered maternal immune factors, intrinsic fetal susceptibility factors also play important roles in disturbed neuroimmune crosstalk through the placenta. This can lead to chronic fetal microglial activation, which may ultimately result in altered neurogenesis, synapse formation, and pruning. In our study, a higher prevalence of childhood tics and Tourette's disorder resulted from overall maternal autoimmune disease, especially in mothers with rheumatoid arthritis, systemic lupus erythematosus, Sjogren's syndrome, ankylosing spondylitis, Graves’ disease, Hashimoto's thyroiditis, and type I diabetes. The results indicate that the maternal autoimmune diseases triggered immune-inflammatory processes involved in the development of tics and Tourette's disorder in the offspring.

Aside from these autoimmune diseases, our study showed that children born to mothers with urinary tract infections, diabetes mellitus, hyperlipidemia, anemia, sleep disorder, endometriosis, and depression had a higher risk of tics and Tourette's disorder (Supplement Table S1), which implies that infections, metabolic conditions, dysbiosis-induced gut-brain axis, and emotional or environmental stress could explain the neuroinflammatory condition facilitating tics and Tourette's disorder, as noted in previous studies (11, 33, 68). A higher prevalence was noted in boys and children with congenital defects in our study, suggesting crucial roles for hereditary, genetic, or epigenetic factors in the development of tics and Tourette's disorder. Interestingly, depressed fathers appear to somehow play a role in the neurobehavioral disorders of their offspring, while other paternal factors showed no association, which serves as a reminder that postnatal education, environment, emotional stress, and poor communication may also contribute to this neurodevelopmental disorder.

The study's strengths include its large sample size and long follow-up period. The availability of comprehensive historical medical service records for all cases has enabled us to mitigate potential selection or recall biases, ensuring a robust evaluation of our hypothesis. However, there were still some potential limitations in our study. First, the NHIRD lacks data on various covariates, including personal lifestyle, family history, education status, social adversity, laboratory data, genetic sequencing, and environmental factors. Despite our efforts to adjust for several comorbidities and match propensity scores, the presence of these unmeasured confounding factors may have potentially impacted our results. Second, the diagnoses of maternal autoimmune diseases, childhood tics, Tourette's disorder, and comorbidities depend on the ICD codes in the dataset. A comprehensive review of all medical records was not conducted to confirm the accuracy of the diagnoses, potentially leading to some instances of misclassification. Because of this, we did not categorize the outcome into different kinds of tics or Tourette's disorder. That is, patients seeking medical care exhibiting similar symptoms or such abnormal behaviors, who had three outpatient department visits or one admission record with any type of tic or Tourette's disorder, were enrolled as the outcome in this study. Second, we do not know whether the underlying maternal autoimmune diseases were active during pregnancy, which may also influence or strengthen the results. Third, neurological autoimmune disorders such as multiple sclerosis were not included in the study. As a study suggested that maternal multiple sclerosis is not associated with a higher risk of neurodevelopmental disorders in offspring, perhaps due to the organ-specific characteristic of this CNS immune-mediated disease, there may be minimal influence on our study results (26). Moreover, it 's important to note that the vast majority of these patients were residents of Taiwan, predominantly of Asian ethnicity. As a result, the applicability of these findings to other countries or ethnic groups may require careful consideration. Finally, it is worth highlighting that the precise mechanisms through which maternal autoimmune diseases might impact the risk of Tourette's and tic disorders were not explored in this analysis, indicating a need for further research. Exploring the association between Tourette's disorder, neurodevelopmental disorders, and maternal autoimmune disorder would also have additional synergistic value to this research topic.

In conclusion, maternal autoimmune diseases can be considered a risk factor for developing tics and Tourette's disorder in offspring, especially in mothers with rheumatoid arthritis, system lupus erythematosus, Sjogren's syndrome, ankylosing spondylitis, Graves’ disease, Hashimoto's thyroiditis, or type I diabetes. We hope that the epidemiological observational results presented in this study can raise clinicians’ awareness of the relationship between tics and Tourette's disorder in offspring and maternal autoimmune diseases, as early diagnosis and treatment can improve outcomes for individuals with tics and Tourette's disorder. Further research is warranted to investigate the possible pathogenetic mechanisms of this association.

## Data Availability

The datasets presented in this study can be found in online repositories. The names of the repository/repositories and accession number(s) can be found below: the LHID is a subset of the NHIRD, a database of all medical claims in Taiwan's NHI system. The usage of NHIRD is limited to research purposes only. Only Taiwanese citizens who fulfill the requirements for conducting research projects are eligible to apply for access to the National Health Insurance Research Database (NHIRD). Applicants must follow the Personal Data Protection Act (https://law.moj.gov.tw/ENG/LawClass/LawAll.aspx?pcode=I0050021) and related regulations of the National Health Insurance Administration and NHRI (National Health Research Institutes), and an agreement must be signed by the applicant and his/her supervisor upon application submission. The datasets generated and analyzed during the current study are available from the authors on reasonable request.
