# Supplementary material for: Maternal autoimmune diseases and the risk of tics and Tourette's disorder in offspring: insights from Taiwan's real-world data
Source: Front Pediatr. 2025 Mar 4;13:1440366. doi: 10.3389/fped.2025.1440366 (PMC11913676; doi:10.3389/fped.2025.1440366)
Supplement: Supplementary file 1 [file Table1.docx]

**Supplementary Table 1. aHRs for offspring’s Tics disorder by multiple Cox regression**

|  | aHR | 95% CI | p value |
| --- | --- | --- | --- |
| Maternal autoimmune disease | 1.22 | 1.15-1.29 | <.0001 |
| **Offspring Factors** |  |  |  |
| Birth year |  |  |  |
| 2009 | Reference |  |  |
| 2010 | 1.04 | 0.97-1.11 | 0.246 |
| 2011 | 1.16 | 1.09-1.24 | <.0001 |
| 2012 | 1.27 | 1.19-1.36 | <.0001 |
| 2013 | 1.35 | 1.26-1.46 | <.0001 |
| 2014 | 1.55 | 1.43-1.67 | <.0001 |
| 2015 | 1.61 | 1.47-1.77 | <.0001 |
| 2016 | 1.97 | 1.76-2.20 | <.0001 |
| Gender |  |  |  |
| Male | Reference |  |  |
| Female | 0.43 | 0.42-0.45 | <.0001 |
| Gestational weeks |  |  |  |
| <37 | 0.90 | 0.85-0.96 | 0.0011 |
| 37-41 | Reference |  |  |
| >=41 | 1.13 | 1.03-1.24 | 0.0092 |
| Birth weight (grams) |  |  |  |
| <2500 | 1.09 | 1.03-1.17 | 0.0068 |
| 2500-3500 | Reference |  |  |
| >=3500 | 0.90 | 0.86-0.94 | <.0001 |
| Congenital defects | 1.15 | 1.06-1.25 | 0.0007 |
| **Maternal Factors** |  |  |  |
| Age (years) |  |  |  |
| <30 | 0.97 | 0.94-1.01 | 0.1541 |
| 30-39 | Reference |  |  |
| >=40 | 0.97 | 0.88-1.08 | 0.6055 |
| Urbanization |  |  |  |
| Urban | Reference |  |  |
| Sub-urban | 0.93 | 0.90-0.96 | <.0001 |
| Rural | 0.783 | 0.72-0.85 | <.0001 |
| Insurance Unit |  |  |  |
| Government | 1.03 | 0.98-1.09 | 0.2724 |
| Labor | Reference |  |  |
| Agricultural/fisherman/water resources employee | 0.79 | 0.72-0.86 | <.0001 |
| Low-income | 0.33 | 0.17-0.64 | 0.0009 |
| Non-labor force | 0.77 | 0.72-0.82 | <.0001 |
| Others | 0.99 | 0.91-1.08 | 0.8028 |
| Delivery methods |  |  |  |
| NSD | Reference |  |  |
| C/S | 1.00 | 0.97-1.04 | 0.7853 |
| Parity |  |  |  |
| Singleton | Reference |  |  |
| Multiparity | 0.97 | 0.88-1.06 | 0.4672 |
| Comorbidity while pregnancy |  |  |  |
| Asthma | 1.09 | 0.95-1.24 | 0.2343 |
| Hypertension | 0.87 | 0.75-1.00 | 0.0425 |
| Diabetes mellitus | 1.05 | 0.96-1.14 | 0.2965 |
| Hyperlipidemia | 1.23 | 1.03-1.48 | 0.0244 |
| Malignancy | 1.12 | 0.88-1.42 | 0.3737 |
| UTI | 1.11 | 1.06-1.16 | <.0001 |
| Seizure disorder | 0.99 | 0.66-1.49 | 0.9648 |
| Anemia | 1.01 | 0.95-1.06 | 0.8634 |
| Gestational diabetes | 1.05 | 0.99-1.10 | 0.1221 |
| Eclampsia or preeclampsia | 1.06 | 0.98-1.15 | 0.1292 |
| Endometriosis | 1.27 | 1.12-1.44 | 0.0001 |
| Sleep disorder | 1.13 | 1.06-1.22 | 0.0003 |
| Depression | 1.04 | 0.95-1.14 | 0.4357 |
| Postpartum depression | 1.20 | 1.12-1.29 | <.0001 |
| Medications during pregnancy |  |  |  |
| Aspirin | 1.15 | 1.04-1.26 | 0.0046 |
| Hydroxychloroquine | 1.36 | 1.05-1.75 | 0.0195 |
| Methotrexate | 0.61 | 0.15-2.47 | 0.4864 |
| Azathioprine | 0.63 | 0.33-1.21 | 0.1669 |
| Cyclosporin | 0.70 | 0.17-2.82 | 0.6124 |
| Sulfasalazine | 1.11 | 0.61-2.04 | 0.7333 |
| Death within 1 year of delivery | 0.59 | 0.19-1.84 | 0.3635 |
| **Paternal Factors** |  |  |  |
| Age |  |  |  |
| <30 | 0.96 | 0.92-1.00 | 0.0505 |
| 30-39 | Reference |  |  |
| >=40 | 0.95 | 0.90-1.00 | 0.0424 |
| Comorbidity |  |  |  |
| Asthma | 1.11 | 0.99-1.25 | 0.0676 |
| Hypertension | 0.99 | 0.91-1.09 | 0.8874 |
| Diabetes mellitus | 0.89 | 0.77-1.02 | 0.0958 |
| Hyperlipidemia | 1.08 | 1.00-1.17 | 0.0627 |
| Malignancy | 1.06 | 0.84-1.34 | 0.6112 |
| UTI | 1.07 | 0.95-1.22 | 0.2754 |
| Seizure disorder | 0.98 | 0.66-1.43 | 0.8974 |
| Sleep disorder | 1.06 | 0.99-1.14 | 0.0913 |
| Depression | 1.18 | 1.08-1.28 | 0.0001 |
| Death within 1 year of delivery | 0.89 | 0.49-1.60 | 0.6855 |

NSD: Normal spontaneous delivery.

C/S: Caesarean section
